# A Compact Multiplexed Hazardous Gas Sensing Platform for Real-Time Monitoring in Mining Scenarios

**DOI:** 10.1021/acsmeasuresciau.6c00033

**Published:** 2026-04-09

**Authors:** Diandra Nunes Barreto, Danielle da Silva Sousa, Lisa Walter, Renan Kobal de Oliveira Alves Cardoso, Fahd Al-Seba’ey, David Gache, João Flávio da Silveira Petruci, Boris Mizaikoff

**Affiliations:** † 9189 Ulm University, Institute of Analytical and Bioanalytical Chemistry, Ulm 89081, Germany; ‡ Federal University of Uberlândia (UFU), Institute of Chemistry, Uberlândia, MG 38400-902, Brazil; § Alpes Lasers, Avenue des Pâquiers 1, St-Blaise 2072, Switzerland; ∥ São Paulo State University (UNESP), Institute of Chemistry, Araraquara, SP 14800-060, Brazil

**Keywords:** laser-based spectroscopy, mid-infrared, quantum cascade lasers, substrate-integrated hollow waveguides, miniaturization, real-time sensors

## Abstract

The determination of hazardous gases in confined environments, such as underground mines, is essential for ensuring suitable occupational health conditions. Although gas chromatography coupled with mass spectrometry (GC-MS) is the gold-standard analytical technique for gas analysis, its application is not feasible in this context due to the bulkiness of the instrumentation and the inability to provide real-time responses. Alternative low-cost sensing techniques, such as metal oxide semiconductors (MOXs), are limited by low molecular selectivity, susceptibility to humidity interference, and long-term instability. Herein, we propose an integrated and compact analytical platform that combines multiple quantum-cascade lasers (QCLs) with substrate-integrated hollow waveguides (iHWGs) acting as miniaturized gas cells for the real-time quantification of hazardous gases, including SO_2_, H_2_S, CO_2_, CH_4_, NO, and NO_2_, in confined workspaces such as underground mines. Each QCL was selected and tuned to match the absorbance profile of a particular target analyte, with the exception of H_2_S, which was converted to SO_2_ via UV irradiation prior to quantification. An Arduino-based system facilitates signal acquisition and processing, enabling rapid data interpretation and wireless communication with external devices. At optimized conditions, the system enables the quantification of all target gases near their permissible exposure limits (PELs) in underground mines, with limits of detection of 0.3, 7, 100, 100, 5, and 3 ppmv for SO_2_, H_2_S, CO_2_, CH_4_, NO, and NO_2_, respectively. The entire system has a footprint of 470 × 320 × 100 mm, which is compatible with real-world deployment scenarios in underground mines. This advanced sensing platform provides real-time monitoring of toxic gases in confined environments with high molecular selectivity and suitable sensitivity. Designed for harsh conditions, it can operate reliably at high humidity, dust, vibrations, and electromagnetic interferences.

## Graphical abstract

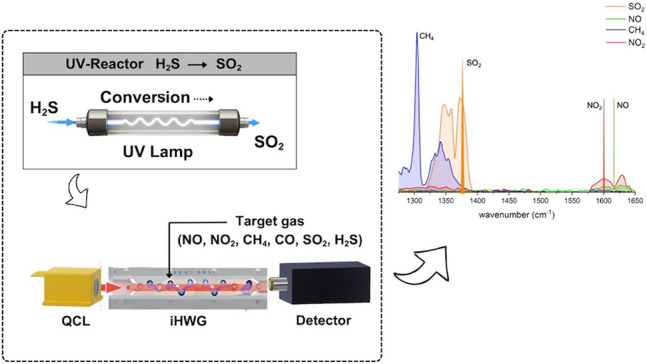

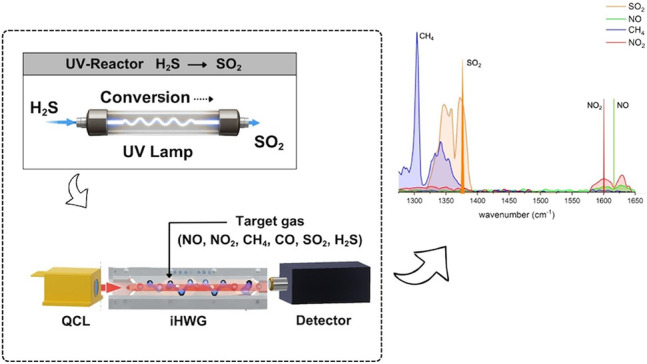

## Introduction

People, especially in industrialized nations, usually spend approximately 90% of their lifetime in indoor environments.^1^ Therefore, the indoor air quality is a critical factor in confined spaces, such as schools, workplaces and homes. Several gases are produced and released to indoor environments, such as, but not limited to methane (CH_4_), hydrogen sulfide (H_2_S), sulfur dioxide (SO_2_), nitrogen dioxide (NO_2_), nitrogen oxide (NO), and carbon dioxide (CO_2_).^1,2^ Due to the toxic effects of some of these gases combined with confined and low ventilated spaces, their concentration can rapidly increase, making indoor locations particularly hazardous.

Combustion processes are the primary sources of gases such as CH_4_, CO_2_, NO, NO_2_ and SO_2_, particularly when fossil fuels are used. At certain conditions, H_2_S can also be generated and released into indoor environments, contributing to both malodor and air toxicity.^3^ Underground mines exemplify particularly unhealthy workplaces, as they amplify the risks of exposure to toxic gases due to confined spaces, high humidity, poor ventilation, and multiple combustion sources including engines, pumps, and other fuel-driven equipment.^4–6^ The mining sector plays a vital role in the global economy, supplying essential raw materials to numerous industries.^7^ According to the International Energy Agency (IEA), the need for critical minerals such as lithium and cobalt is projected to rise by up to 500% by 2050 driven by population growth and increasing demand for mineral resources.

To mitigate the risks associated with hazardous gases in workplaces such as underground mines, regulatory agencies such as the Occupational Safety and Health Administration (OSHA), the American Conference of Governmental Industrial Hygienists (ACGIH) and the Mine Safety and Health Administration (MSHA) have established permissible exposure limits (PELs) for the most relevant gases found in such indoor atmospheres from usual sources.^7^ For example, PELs of 5, 10, 5000, 10000, 25, and 5 ppm have been set for SO_2_, H_2_S, CO_2_, CH_4_, NO and NO_2_, respectively.^8^ However, the real-time quantification of multiple gases in these scenarios remains a challenging analytical task, particularly if the analytes are present at concentrations ranging from ppmv to ppbv. In such scenarios, the analytical platform must ensure high molecular selectivity to minimize cross-interference, compact dimensions and operational features suitable for in-field deployment along with adequate sensitivity across a broad range of concentrations and species. Furthermore, water vapor interference must be considered due to the high humidity typically encountered in underground mining environments.

Gas chromatography coupled to mass spectrometry (GC-MS) is widely considered as the “gold standard” technique for gas detection.^9^ However, its bulky dimensions, limitation in operation to laboratory environments, and inability to provide real-time responses limit its applicability when short-term variations in analyte concentrations must be monitored. Metal oxide semiconductor (MOX)-based sensors have been used for gas analysis with the advantages of low cost and rapid response. Nevertheless, their performance is limited by the need for elevated operating temperatures, strong interference from air humidity, and susceptibility to cross-interference.^10^ On the other hand, optical sensors operating in the mid-infrared (MIR; 2.5–25 μm) spectral range offer highly specific molecular discrimination via the excitation of fundamental vibrational and rovibrational transitions.^11^ IR sensors based on conventional Fourier transform infrared (FTIR) spectroscopy have been successfully employed for the quantification of the aforementioned gases with suitable selectivity and sensitivity.^3,12–14^ However, most implementations rely on complex gas cells providing suitably extended absorption path lengths and bulky spectrometers.^15^

Substrate-integrated hollow waveguides (iHWGs) are a recently developed generation of gas cells, pioneered by the research group of Mizaikoff and collaborators.^16^ Compared to traditional multipath White or Herriott cells, these devices consist of two highly polished metallic substrates with compact dimensions, one of which contains a hollow channel structure that facilitates a designable and adaptable optical path length. When integrated with the other planar substrate, IR radiation is guided through the channel via multiple reflections. By injecting gaseous samples into the same hollow channel, iHWGs simultaneously function as an extremely low-volume gas cell with a well-defined optical absorption path length and an exceptionally short sample residence time, thus facilitating miniaturized yet rapidly responding MIR gas-sensing strategies. Since their introduction, the combination of FTIR spectroscopy and iHWGs has been applied to the monitoring of a wide range of gases including but not limited to H_2_S, SO_2_, NO_*x*_, CH_4_, and O_3_.^3,12,17,18^

Quantum cascade lasers (QCLs) are unipolar semiconductor devices that operate via electronic transitions within a single conduction or valence band. They have been employed as light sources for developing advanced integrated MIR sensors due to their high output power and narrow emission bandwidth.^19,20^ Additionally, QCLs can be operated using tailored driving electronics controlled by microcontrollers (e.g., Arduinos), which expands their ease of use and level of integration in routine applications. The combination of QCLs and iHWGs intrinsically reduces the footprint of the gas sensing platform, enabling portability and deployment for in-field gas monitoring scenarios, as fundamentally demonstrated by our research team.^21–23^ However, the use of a multi-QCL-multi-iHWG sensing platform for multianalyte quantification in enclosed environments—particularly at high humidity and dust conditions—has not been demonstrated to date.

Consequently, in this study we have developed a portable optical sensor platform based on the integration of multiple QCLs with multiple iHWGs facilitating online and on-site quantification of SO_2_, H_2_S, CO_2_, CH_4_, NO and NO_2_ within concentration ranges near to the PELs. Lasers and detectors were operated using an Arduino-based code, providing remote control and data transmission to mobile devices. The performance metrics of the developed sensing platform were validated ensuring suitability for future monitoring of hazardous gases in workplace environments such as underground mines.

## Experimental Section

Gas mixtures containing sulfur dioxide, hydrogen sulfide, nitrogen oxide, nitrogen dioxide, methane, carbon dioxide, nitrogen and synthetic air were obtained from MTI IndustrieGase AG (Neu-Ulm, Germany). A gas mixing prototype based on mass flow controllers (Bronkhorst High-Tech BV, Netherlands) developed by the Institute of Analytical and Bioanalytical Chemistry at the University of Ulm and Lawrence Livermore National Laboratory (LLNL, Livermore, USA) was used for preparing and delivering gaseous standards at varying concentrations dispersed in nitrogen or synthetic air at the desired flow rate (i.e., up to 200 mL min^–1^). The UV-assisted H_2_S–SO_2_ conversion was performed by exposing H_2_S–synthetic air mixtures to ultraviolet radiation at 185 nm using a custom quartz tube flow device with the tubular conversion cell coiled around a miniaturized UV lamp (UV-C, Rexim LLC, Watertown, MA, USA) with dimensions of 47 × 6 × 47 mm; length × width × depth, as previously reported.^17^ The lamp is supplied with a voltage of 5 V, and a current of 30 mA, thus providing an energy output of 49 μW cm^–2^ at 185 nm.

Distributed feedback—quantum cascade lasers (DBF-QCLs) were provided by Alpes Lasers (Switzerland) with centered emission wavelength of 1373 cm^–1^ (SO_2_ and H_2_S), 2307 cm^–1^ (CO_2_), 1307 cm^–1^ (CH_4_), 1642 cm^–1^ (NO), 1603 cm^–1^ (NO_2_), matching the absorption spectra of the target analytes. Thermoelectrically cooled mercury–cadmium–telluride (MCT) photovoltaic detector modules (LabM-I-5, Vigo Photonics, Poland) were used to detect the IR radiation. The spectral characteristics of each QCL were analyzed by coupling into a high-resolution FTIR spectrometer (model Vertex 80v, Bruker Corporation, Germany) prior to integration into the sensing and operation within the QCL-iHWG sensing system. A low-noise current power supply, 0.4 μA RMS, 100 kHz (model QCL 500 OEM, Wavelength Electronics, Bozeman, MT, USA) and a temperature controller coupled with Peltier-based systems, (model TEC 1161, Laser Diode Technologies, Houston, Texas, USA), were employed to ensure precise current and thermal stabilization during the laser operation and measurements.

Straight-line iHWGs made from polished aluminum providing an integrated hollow waveguide channel (i.e., absorption path length of 12 cm) presented physical dimensions of 160 × 60 × 45 mm (length × width × height) and simultaneously served as miniaturized gas cells. When integrated with the QCL and detector, the complete sensing platform measured 235 × 60 × 85 mm (length × width × height). Both ends of the iHWG were designed to precisely accommodate the optical characteristics of the laser source and detector, ensuring alignment and efficient radiation coupling without additional optical components. Luer locks adapters were positioned on top of the iHWG to allow the introduction of gas samples. Current and temperature drives were integrated into the system and controlled using a laptop or a smartphone. All components were attached to an aluminum breadboard (Thorlabs, Dachau, Germany); one of the several QCL-iHWG sensing channels is schematically shown in Figure 1.

**Figure 1:**
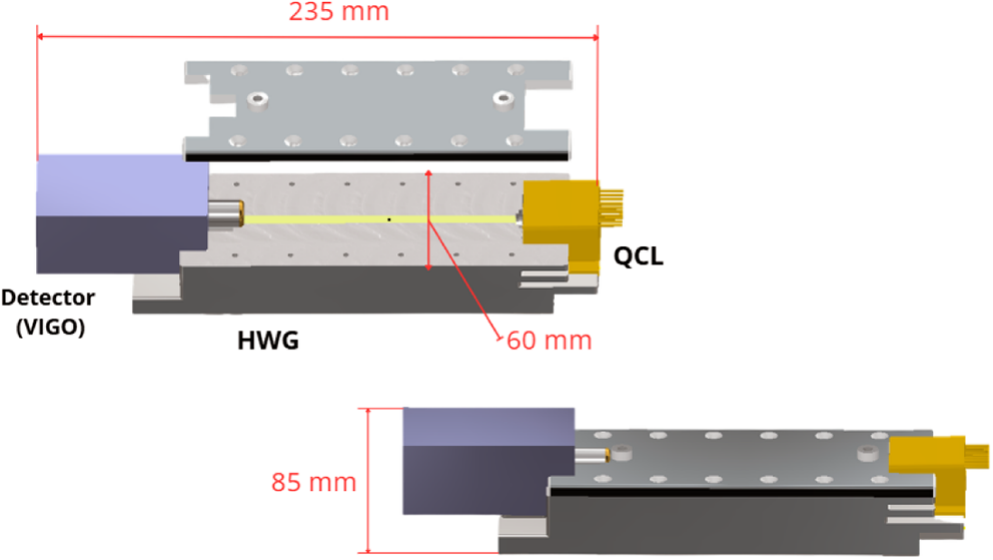
3D rendered model of a single integrated QCL-iHWG channel. This structure is then multiplexed according to the number of target gases to be detected.

## Results and Discussion

### Optimization of the UV-Assisted Conversion of H_2_S to SO_2_

Among the monitored gases (i.e., CO_2_, CH_4_, NO_2_, H_2_S, NO and SO_2_), hydrogen sulfide is the weakest absorber within the MIR spectral range. Therefore, direct quantification at trace concentration levels is difficult even using multipass gas cells. An elegant approach has been previously described by our research team involving the UV-assisted conversion of H_2_S to SO_2_, a strong IR absorber.^17^ The conversion of H_2_S into SO_2_ involves the formation of a hydroxyl radical by the irradiation of light at 185 nm. Previously, we have employed a conventional UV lamp (length: 210 mm) containing a customized coiled quartz tube flow device with length of 95 cm. In a novel approach, four miniaturized UV lamps (47 × 6 × 47 mm; L × W × D) were integrated into two blocks of aluminum and used as an in-flow gaseous conversion system.^24^ In the present study, we have merged both concepts to minimize the footprint of the conversion device by using a coiled quartz tube with small dimensions (11 cm) around a miniaturized UV lamp. Figure 3a shows an image of the UV-assisted conversion module.

To evaluate the efficiency of the H_2_S/SO_2_ conversion, 500 ppm of H_2_S prepared in synthetic air was introduced into the conversion module at airflows ranging from 10 to 30 mL min^–1^ followed by IR measurements. All IR spectra were recorded in the range of 4000–650 cm^–1^ at a spectral resolution of 4 cm^–1^ using a benchtop FTIR spectrometer (Alpha, Bruker, Germany) for comparability equipped with the same thermoelectrically cooled mercury–cadmium–telluride detector then integrated into the sensing platform. Like in the QCL-based sensing system, a straight-line iHWG with an absorption path length of 15 cm was coupled to the optical system. A concept of the device is schematically illustrated in Figure 2b. The conversion rate depends on the flow rate, light path, and power stability, whereas humidity and matrix effects are expected to be negligible, as discussed in previous studies.^3,24^ An airflow of 10 mL min^–1^ was determined the most efficient flow rate to efficiently produce SO_2_ achieving a conversion yield of 54 ± 1%. The conversion yield obtained with the device presented an uncertainty lower than 2%, with negligible impact on the analytical method, and was used during further experiments.

**Figure 2:**
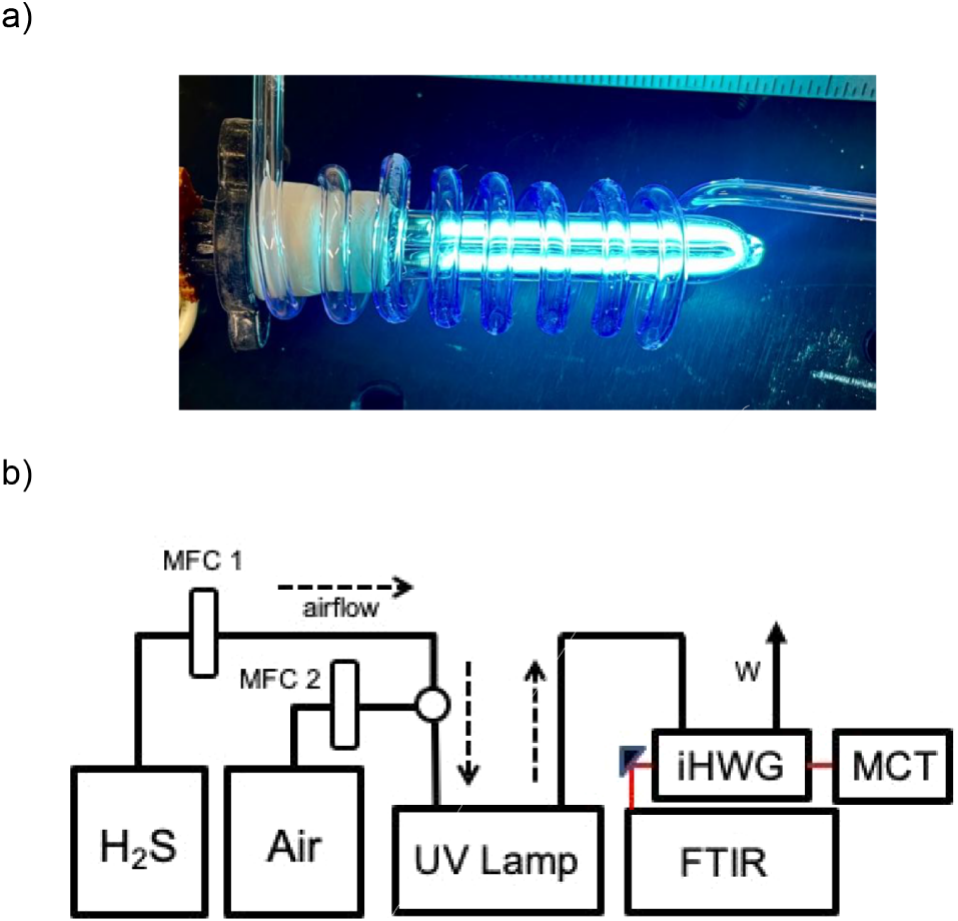
(a) Image of the miniaturized UV-assisted reactor for the in-flow conversion of H_2_S to SO_2_; (b) Conceptual scheme of the integration of the conversion module with an optical detection system for SO_2_.

### Optimization of QCL Operation Conditions

The optical emission characteristics (i.e., wavelength and optical power) of DFB-QCLs are strongly influenced by operational parameters, including applied current and temperature. Controlling these parameters is essential, as the selection of the emitted wavelength and the stability of the output signal directly affect the selectivity and sensitivity of the analytical method. In a first step, the emission wavelength of each QCL as a function of temperature and current was determined by coupling the emitted radiation into an FTIR spectrometer, as detailed in the Experimental Section. Initially, the power stability of each QCL was evaluated over 5 min of continuous operation. Relative standard deviations below 0.5% were obtained, demonstrating the stability of the light sources. Subsequently, the performance of each QCL was evaluated by monitoring optical power and baseline noise after 1 h of continuous operation in a blank air matrix. Signal-to-noise ratios (SNR) were then calculated for an analyte concentration of 0.5% of CO_2_, SO_2_, CH_4_, NO and NO_2_. The gas samples were injected into the optical detection system at an airflow of 100 mL min^–1^. For each analyte, the selected emission wavelength corresponded to the most representative absorption region in the mid-infrared spectrum ensuring adequate selectivity, as shown in Figure 3. At optimized operating conditions, the obtained noise values ranged from 15 to 25 μV, with SNR values between 71 and 120. Table 1 summarizes the optimized current and temperature settings for each QCL in relation to the target analyte.

**Table 1:** Optimized Operational Conditions and Performance Evaluation for Each QCL/Analyte Combination

	CH_4_	CO_2_	SO_2_/H_2_S	NO_2_	NO
Wavenumber (cm^–1^)	1307	2300	1373	1600	1639.7
Wavelength (μm)	7.6	4.3	7.2	6.2	6.1
Temperature (°C)	20	20	25	35	20
Current (mA)	395	283	307	215	130
Output power (mW)	10	3.9	14.4	14.9	8.2
Noise (μV)	19.7	23.4	16.8	13.2	18.1
SNR*	120	111	81	71	52

**Figure 3:**
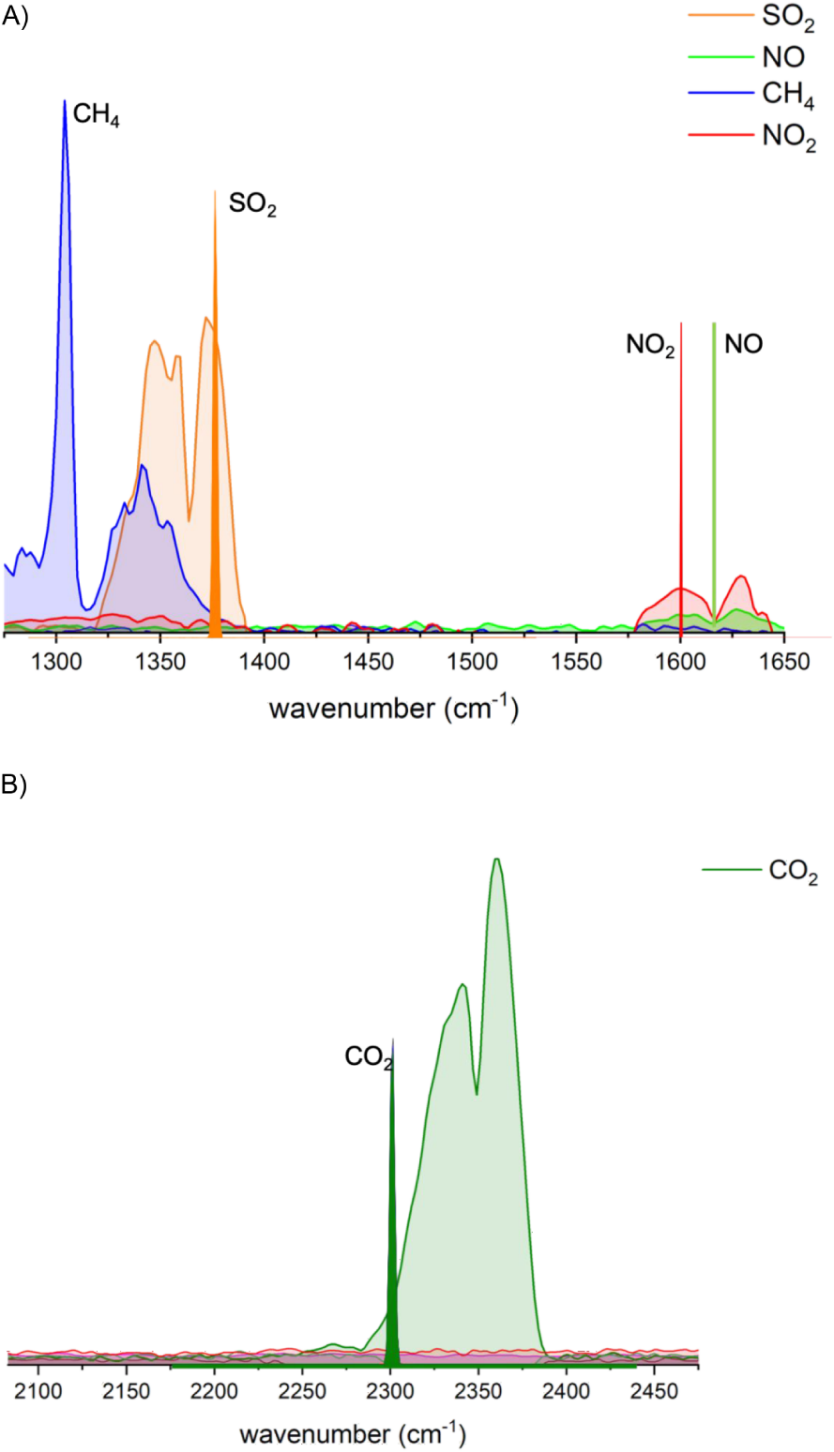
Laser emission lines overlaid with representative IR spectra of the target analytes. (a) SNO_2_, NO_2_, and NO in the 1250–1650 cm^–1^ region. (b) CO_2_ in the 2100–2450 cm^–1^ region.

Potential spectral interferences may arise mainly from water vapor and other infrared-active gases whose absorption bands occur near the selected wavenumbers. In the spectral regions employed in this work, species such as NH_3_, CO, and light hydrocarbons may contribute to partial spectral overlap. Nevertheless, the use of narrow spectral windows minimizes cross-sensitivity effects. Also, the expected concentrations of these gases in air samples are typically in the ppbv range, which further minimizes potential interference.

Additionally, water vapor is not expected to interfere with the optical measurement or affect the iHWG component, as demonstrated in previous work.^25^ Dedicated filters can be installed at the air inlet to prevent light scattering or blockage caused by particulate matter. Due to the direct coupling of the QCL, iHWG, and detector, small vibrations are unlikely to cause optical interference during measurements. Therefore, the proposed analytical platform is expected to operate reliably in real-world environments, such as underground mines.

### Analytical Figures-of-Merit

The analytical platform is based on the combination of multiple QCL-iHWG channels each tailored for the determination of one of the most common gases found in underground mines. With this in mind, the analytical performance was evaluated according to the ability to detect a specific analyte within the required PEL range. The analytical signal (i.e., absorbance) was calculated from the logarithm ratio of the sample and blank averaging the voltage signals obtained at the detector during 1 min. Each gas was injected at an airflow of 100 mL min^–1^, with exception of CO_2_ analyzed at a flow rate of 200 mL min^–1^ to ensure rapid and uniform sample replacement within the absorption cell and H_2_S at a flow rate of 10 mL min^–1^ to maximize residence time in the UV-assisted conversion section. For quantitative purposes, calibration functions were established based on the absorbance values versus the analyte concentration. For each concentration, an average value of three replicate measurements was obtained. The proposed setup revealed excellent linearity (r² > 0.99) across the investigated concentration ranges for each gaseous analyte. Limits of detection (LOD) and quantification (LOQ) for each analyte were obtained as three and ten times the standard deviation of the blank signal divided by the slope of the calibration curve, respectively. The obtained analytical figures-of-merit are summarized in Table 2.

**Table 2:** Analytical-Figures-of-Merit Obtained for the Multiplexed QCL-iHWG Gas Sensing System for the Evaluated Target Gases

	CH_4_	CO_2_	SO_2_	H_2_S	NO	NO_2_
Linear range (ppmv)	100–10000	1000–10000	5–90	10–100	7–90	8–66
Equation	A = 2.3 × 10^–5^ [CH_4_] + 0.007	A = 1.6 × 10^–5^ [CO_2_] + 0.001	A = 4.7 × 10^–5^ [SO_2_] + 0.005	A = 3.4 × 10^–5^ [H_2_S] + 0.006	A = 3.6 × 10^–5^ [NO] – 0.002	A = 4 × 10^–6^ [NO_2_] + 0.0003
r^2^	0.991	0.998	0.998	0.998	0.999	0.999
LOD (ppmv)	100	100	0.3	7	0.9	1.3
LOQ (ppmv)	250	1000	0.9	21	2.7	4.0

The analytical features and performance of the proposed platform were compared with other optical approaches for direct gas analysis. As shown in Table 3, the LOD achieved in this study is comparable to values reported in the literature. The combination of a dedicated QCL, iHWG, and detector contributes to a highly miniaturized platform while still enabling the detection of multiple gases, with no need of preconcentration or sample preparation. Although MOX-based gas sensors are often considered attractive due to their simplicity and fast response, their strong dependence on relative humidity and temperature limits their applicability in real-world scenarios.^10^

**Table 3:** Comparison among Other Optical Analytical Platforms Employed for the Detection of the Target Gases

Sensing approach	Target Gas	LOD	Remarks	Ref.
NDIR	NO_2_	2.8 ppmv	Use of narrow band-pass filters	^26^
FTIR/iHWG	H_2_S/SO_2_	0.2/0.07 ppmv	Preconcentration	^17^
FTIR/iHWG	NO/NO_2_/N_2_O	10/1/0.5 ppmv	Conventional benchtop FTIR	^12^
On-chip MID-Infrared chalcogenide waveguide sensor	CH_4_	2.5 vol %	Waveguide prepared via UV lithography and lift-off/thermal evaporation	^27^
MID-Infrared DFB laser + HWG	CO_2_	3 ppmv	Tubular HWG with collimated lens	^28^
MID-Infrared dual-gas sensor	CO_2_/CO	5.66/0.94 ppmv	Multipass gas cell	^29^
MID-Infrared QCL multipass absorption sensor	SO_2_	8 ppbv	High temperature multipass gas cell	^30^
ICL/iHWG	CH_4_	28 ppmv	Nitrogen-cooled MCT detector	^22^
Multi QCL-iHWG	CO_2_ /CH_4_/H_2_S/SO_2_/NO/NO_2_	100/100/7/0.3/5/3 ppmv	Compact and multiplexed	This work

## Conclusions

An integrated and compact multigas sensing platform combining multiple quantum cascade lasers with substrate-integrated hollow waveguides (QCL-iHWG) was developed and its performance validated, enabling real-time quantification of CH_4_, CO_2_, SO_2_, H_2_S, NO and NO_2_. The system provides multigas detection capabilities with rapid response times (i.e., 1 min) and sufficient sensitivity at hazardous concentration levels defined by permissible exposure limit (PEL) regulations in underground mines. Methane, carbon dioxide, sulfur dioxide, and nitrogen dioxide were directly detected via their characteristic mid-infrared absorption bands, while hydrogen sulfide was indirectly quantified after UV-assisted conversion to SO_2_. The platform was tested across concentration ranges near the PELs of each analyte, confirming its utility and robustness for future in-field toxic gas monitoring in occupational settings with a specific focus on underground mines. For NO_2_, the lower limit of the linear range (8 ppmv) is close to the respective PEL (5 ppmv), while the LOD (1.3 ppmv) remains below this value. In this case, the linear range could not be extended due to limitations associated with the preparation of the gas standard mixtures. Future work will focus on further miniaturization by integrating multiple QCLs into a specifically tailored single iHWG, thereby enabling sequential analysis of gas mixtures and further enhancing the compactness of the developed hazardous gas sensing scheme.
